# Special Issue: New Findings of MXenes: Preparation, Properties and Applications in Biotechnology and Catalysis

**DOI:** 10.3390/ma14040892

**Published:** 2021-02-13

**Authors:** Agnieszka M. Jastrzębska, Jarosław Woźniak

**Affiliations:** Faculty of Materials Science and Engineering, Warsaw University of Technology, Wołoska 141, 02-507 Warsaw, Poland; jaroslaw.wozniak@pw.edu.pl

The discovery of graphene drove intensive studies towards novel two-dimensional (2D) materials [1]. These include recently discovered MXenes, also known as early transition metal carbides and/or nitrides [2]. Their formula *M_n_X_n-1_* (where *M* is an early transition metal and *X* is carbon or nitrogen) reflects a nano-layered structure in which the *X* layer is sandwiched from both sides by the *M* layer [3]. Since MXenes’ discovery in 2011, they have properties that make them promising for applications in biology and medicine, including biosensors [4]. In the case of biotechnological applications, delaminated 2D MXenes flakes are used solo or incorporated via self-assembly and related physicochemical interactions. When considering functional nanocomposite structures, various external stimuli blending approaches are used, for which obtaining the most efficient dispersion is a challenge.

MXenes seem to be the best choice for both abovementioned approaches because of their excellent dispersibility in an impressive number of both polar and non-polar solvents [5], as well as their unique and tunable surface reactivity [6]. Consequently, they have received growing interest in recent years. It is particularly noteworthy that MXenes are currently catching up with the graphene peloton and they are expected to reach technological maturity, as an efficient 2D alternative to graphene, at an unprecedented pace.

The Special Issue “New Findings of MXenes: Preparation, Properties and Applications in Biotechnology and catalysis” is aimed at gathering new findings of MXenes in one place to reach the audience that is interested in this emerging field. The contributed papers were mostly prepared by a young generation of scientists whose intensive research accelerates rapid development of innovations concentrated around 2D MXenes. Therefore, we cordially present a special collection of scientific works that discuss the use of MXenes as a functional additive to both organic and ceramic composites together with elucidating cytotoxic issues on both theoretical and experimental means.

It is accepted that current trends in the field of MXenes emphasize the importance of controlling their surface features for successful application in biotechnological areas. The corresponding work lead by Anita Rozmysłowska-Wojciechowska et al. [7], entitled “Engineering of 2D Ti_3_C_2_ MXene Surface Charge and its Influence on Biological Properties”, revealed the ability to stabilize the surface properties of MXenes via surface charge engineering. It was shown that the surface charges of 2D Ti_3_C_2_T_x_ MXene flakes can be easily changed by using cationic polymeric poly-L-lysine (PLL) molecules. Because of electrostatic PLL surface-adsorption, both colloidal and biological properties of MXene were changed. The related effects were changing the negative surface charge toward a positive value and its managing through pH changes. Analysis of bioactive properties revealed the presence of biocidal properties against *E. coli* together with cytocompatibility adjustment in vitro at a very high concentration, even up to 375 mg L^−1^. The presented study demonstrates a feasible approach to control surface properties of 2D Ti_3_C_2_T_x_ MXene flakes through surface charge engineering that not only prevents potential cytotoxicity but also allows for future nanomedical applications.

It is noted that due to time-consuming and expensive in vitro and in vivo studies, only part of developed materials can reach the early pre-clinical stages of development. The machine learning methods can be used by extracting new insights from available biological data sets and provide further guidance for experimental studies. The theoretical paper of Maciej E. Marchwiany et al. [8], entitled “Surface-Related Features Responsible for Cytotoxic Behavior of MXenes Layered Materials Predicted with Machine Learning Approach”, continues the issue of MXenes cytotoxicity to speed up the implementation of MXenes in biomedical applications. The study identified various surface-specific features that might be responsible for the cytotoxic behavior of 2D MXenes. These are transition metal oxides and lithium that are present on the surface. Using the identified cytotoxicity-generating features, the authors developed a machine learning model that succeeded in predictioning potential toxicity for 2D MXenes that were not tested in vitro.

A recent discovery of the unique biological properties of two-dimensional transition metal carbides (MXenes) resulted in intensive research on their application in various biotechnological areas, including polymeric nanocomposite systems. The potential of MXene as an additive to bioactive natural porous composite structures is highlighted in the work of Anita Rozmysłowska-Wojciechowska et al. [9], entitled “Controlling the Porosity and Biocidal Properties of the Chitosan-Hyaluronate Matrix Hydrogel Nanocomposites by the Addition of 2D Ti_3_C_2_T_x_ MXene”, which goes one step further in investigating the hydrogel nanocomposites functionalized by 2D MXene. Herein, it is reported that the addition of 2D Ti_3_C_2_T_x_ MXene to hydrogels maintains their desired antibacterial properties by reducing the porosity of the chitosan-hyaluronate matrix nanocomposite structures, additionally stabilized by vitamin C. This was confirmed by micro-computed tomography visualization which enables insight into the porous structure of nanocomposites. It was also found that a small amount of MXene (1–5 wt.%) was effective against gram-negative *E. coli*, gram-positive *S. aureus*, and *Bacillus sp.* bacteria in a hydrogel system. Such an approach unequivocally advances the future design approaches of modern wound healing dressing materials with the addition of MXenes.

The issue of biocidal properties is continued by Michał Jakubczak et al. [10] within a work entitled “Filtration Materials Modified with 2D Nanocomposites—A New Perspective for Point-of-Use Water Treatment”. Point-of-use (POU) water treatment systems and devices play an essential role in limited access to sanitary safe water resources. An innovative, 2D Ti_3_C_2_/Al_2_O_3_/Ag/Cu nanocomposite-modified filtration material is herein reported with the application potential for POU water treatment. The material is characterized by improved filtration velocity relative to unmodified reference material, effective elimination of microorganisms, and self-disinfecting potential, which afforded the collection of 99.6 wt.% of bacteria in the filter. The effect was obtained with nanocomposite levels as low as 1 wt.%. In addition, surface oxidation of the modified material increased its antimicrobial efficiency while no secondary release of the nanocomposites into the filtrate was observed.

The subsequently identified field to grow in the case of MXenes is the surface reactivity, which can be easily tailored for obtaining various surface-modified functional nanocomposite structures. The work of Tomasz Wojciechowski et al. [11], entitled “Non-toxic 2D Ti_3_C_2_ MXene surface-modified with Al, Ga, In alkoxides by chemical reactions with metal trialkyls”, made the case that 2D Ti_3_C_2_ MXene can undergo reactions with organometallic compounds to obtain functional non-toxic nanocomposite structures ready to be applied in biotechnology. Herein, 2D Ti_3_C_2_ MXene-based nanocomposites are presented. They were obtained via the formation of covalent metal–oxygen cross-linking between MXene terminal functional groups and three organometallic compounds (Et_3_Al, Me_3_In, and Et_3_Ga) that are highly reactive toward oxygen and hydroxyl groups. In this regard, the mechanism of the process of surface-bonding of organometallic compounds is both driven and limited by compounds’ reactivity and surface steric conditions. Further microbiological studies toward *S. aureus*, *E. coli*, and *C. albicans* confirmed the absence of acute nano-toxicity for developed nanocomposites. Evaluation of potential inhibition of bioluminescence with the soil bacteria *Photorhabdus luminescens* also did not reveal any ecotoxicological behavior of developed materials.

Preparation of ceramic matrix composites is a great challenge because of the need for efficient dispersion of the strengthening phase as well as obtaining the expected added value at a reasonable amount of incorporated material. The work of Jarosław Woźniak et al. [12], entitled “Influence of MXene (Ti_3_C_2_) Phase Addition on the Microstructure and Mechanical Properties of Silicon Nitride Ceramics”, discusses the influence of Ti_3_C_2_T_x_ (MXene) addition on silicon nitride and its impact on the microstructure and mechanical properties of the latter. Composites were prepared through powder processing and sintered using the spark plasma sintering (SPS) technique. Relative density, hardness and fracture toughness were analyzed. The highest fracture toughness at 5.3 MPa·m^1/2^ and the highest hardness at HV5 2217 were achieved for 0.7 and 2 wt.% Ti_3_C_2_T_x_, respectively. Moreover, the formation of the Si_2_N_2_O phase was observed as a result of both the MXene addition and the preservation of the α-Si_3_N_4_ → β-Si_3_N_4_ phase transformation during the sintering process.

Further work of Tomasz Cygan et al. [13], entitled “Microstructure and Mechanical Properties of Alumina Composites with Addition of Structurally Modified 2D Ti_3_C_2_ (MXene) Phase”, concentrates on structural modifications of MXene to harness its oxidation and degradation, particularly at higher temperatures. This is achieved by creating the metallic layer on the Ti_3_C_2_T_x_, by sputtering the titanium or molybdenum. The powder metallurgy and Spark Plasma Sintering (SPS) technique were adopted for preparation of the composites. The effectiveness of the applied modifications, microstructure, and mechanical properties of the obtained sinters was examined. The performed observations revealed significant changes in the MXenes degradation process, from porous areas with TiC particles (for unmodified Ti_3_C_2_T_x_), to in-situ creation of graphitic carbon (in case of Ti_3_C_2_-Ti/Mo). In this regard, the fracture changed from purely intergranular to cracking with transgranular mode.

Just to summarize, it is now well accepted that a potentially rich area of novel findings can involve MXenes and there is no doubt that this new class of 2D materials will grow rapidly as an innovation-generating field. Some unique properties of MXenes have been already identified and will be further developed, leading to new applications. Nevertheless, it is important to fully understand the synthesis–structure–property relationship corresponding to the chosen application. Understanding the surface chemistry and reactivity of 2D MXenes is of fundamental importance in terms of the development of more advanced functional materials. This cannot be done without combining experimental and theoretical knowledge as well as without the in-depth studies of corresponding material effects. We state that the future directions for research will be highly accelerated by 2D structures beyond graphene (including also MXenes) and their new applications in biotechnology and functional nano-composite structures are to be expected soon.
